# Development of an Electronic Data Collection System to Support a Large-Scale HIV Behavioral Intervention Trial: Protocol for an Electronic Data Collection System

**DOI:** 10.2196/10777

**Published:** 2018-12-14

**Authors:** W Scott Comulada, Wenze Tang, Dallas Swendeman, Amy Cooper, Jeremy Wacksman, Adolescent Medicine Trials Network (ATN) CARES Team

**Affiliations:** 1 Department of Psychiatry and Biobehavioral Sciences University of California, Los Angeles Los Angeles, CA United States; 2 Dimagi Inc Cambridge, MA United States; 3 Adolescent Medicine Section Department of Pediatrics Tulane University New Orleans, LA United States; 4 College of Osteopathic Medicine Nova Southeastern University Fort Lauderdale, FL United States; 5 Department of Family and Community Medicine UT Southwestern Medical Center Dallas, TX United States; 6 Division of Prevention Science School of Medicine University of California, San Francisco San Fransisco, CA United States

**Keywords:** electronic data capture, ecological momentary intervention, HIV prevention and treatment, mHealth, mobile phone, text messaging

## Abstract

**Background:**

Advancing technology has increased functionality and permitted more complex study designs for behavioral interventions. Investigators need to keep pace with these technological advances for electronic data capture (EDC) systems to be appropriately executed and utilized at full capacity in research settings. Mobile technology allows EDC systems to collect near real-time data from study participants, deliver intervention directly to participants’ mobile devices, monitor staff activity, and facilitate near real-time decision making during study implementation.

**Objective:**

This paper presents the infrastructure of an EDC system designed to support a multisite HIV biobehavioral intervention trial in Los Angeles and New Orleans: the Adolescent Medicine Trials Network “Comprehensive Adolescent Research & Engagement Studies” (ATN CARES). We provide an overview of how multiple EDC functions can be integrated into a single EDC system to support large-scale intervention trials.

**Methods:**

The CARES EDC system is designed to monitor and document multiple study functions, including, screening, recruitment, retention, intervention delivery, and outcome assessment. Text messaging (short message service, SMS) and nearly all data collection are supported by the EDC system. The system functions on mobile phones, tablets, and Web browsers.

**Results:**

ATN CARES is enrolling study participants and collecting baseline and follow-up data through the EDC system. Besides data collection, the EDC system is being used to generate multiple reports that inform recruitment planning, budgeting, intervention quality, and field staff supervision. The system is supporting both incoming and outgoing text messages (SMS) and offers high-level data security. Intervention design details are also influenced by EDC system platform capabilities and constraints. Challenges of using EDC systems are addressed through programming updates and training on how to improve data quality.

**Conclusions:**

There are three key considerations in the development of an EDC system for an intervention trial. First, it needs to be decided whether the flexibility provided by the development of a study-specific, in-house EDC system is needed relative to the utilization of an existing commercial platform that requires less in-house programming expertise. Second, a single EDC system may not provide all functionality. ATN CARES is using a main EDC system for data collection, text messaging (SMS) interventions, and case management and a separate Web-based platform to support an online peer support intervention. Decisions need to be made regarding the functionality that is crucial for the EDC system to handle and what functionality can be handled by other systems. Third, data security is a priority but needs to be balanced with the need for flexible intervention delivery. For example, ATN CARES is delivering text messages (SMS) to study participants’ mobile phones. EDC data security protocols should be developed under guidance from security experts and with formative consulting with the target study population as to their perceptions and needs.

**International Registered Report Identifier (IRRID):**

DERR1-10.2196/10777

## Introduction

### Background

Data collection systems are integral to the design of behavioral intervention trials and have rapidly evolved from systems that are limited by rudimentary data collection tools, such as pen-and-paper assessments, to electronic data capture (EDC) systems that incorporate mobile phones and other mobile data collection devices. Advancing technology has increased functionality and permitted more complex study designs. To date, papers on mobile EDC systems have focused on improving health care delivery, disease surveillance, and epidemiological surveys in resource-poor settings [1-6]. Few publications describe the use of mobile EDC systems to support multiple study interventions, study management, as well as outcome assessments [4,7]. This paper fills in gaps in the literature by describing the EDC system from an ongoing HIV biobehavioral intervention trial, Adolescent Medicine Trials Network “Comprehensive Adolescent Research & Engagement Studies” (ATN CARES), and highlights the benefits and limitations of what current EDC systems can provide. We begin with an overview of EDC system functionality and highlight salient features for behavioral intervention trials.

As a basic role, EDC systems support data collection and increase the capacity to collect and store different types of data, compared with non-EDC systems. The ability of EDC systems to link to different mobile devices offers flexibility in capturing information over different time intervals and locations and from different sources. Our prior work has used EDC systems to store biological, anthropometric, social network, and self-reported measures that were collected over several time points as part of large-scale behavioral intervention trials in South Africa [8,9], India [10], and the United States [11]. In addition, EDC systems provide access to data in near real time, often through a Web portal. Easy data access throughout the study implementation period facilitates improved decision making and monitoring by research staff supervising the study progress. Staff activity monitoring in the literature has been presented for resource-poor settings, as in the monitoring of community health workers in Africa [12,13].

The ability to link EDC systems to study participants’ mobile devices provides additional opportunities for intervention delivery, referred to as ecological momentary interventions [14]. EDC systems have been used to deliver automated short message service (SMS) text messages to participants’ mobile phones to provide health education, medication, and clinic visit appointment reminders [15,16]. Frequent reporting through ecological momentary assessment (EMA) [17-19] is a mechanism for self-reflection and self-management that can lead to behavior change on its own, referred to as “reactivity” [17,18,20]. Lastly, EMA data collection and the ability to view EDC system data in near real time provides opportunities to intervene in EMA responses in a timely manner relative to less frequent reporting that occurs during in-person follow-up visits.

The EDC system for ATN CARES incorporates multiple functions summarized above: data collection, mobile phone-based intervention delivery, and real-time data access for timely data quality monitoring and decision making that pertains to participant care. Most mobile EDC systems utilized in the past did not have multiple functions [21]. The ATN CARES population also provides opportunities to highlight the benefits and limitations of current mobile EDC systems.

Study participants are youth living with HIV (YLH) and HIV-negative high-risk youth (HRY). By focusing on YLH and HRY, we highlight the benefits of what an EDC system can provide for a tech-savvy study population that already uses mobile phones to a high degree in its daily routines [22]. In addition, a focus on YLH and HRY underscores considerations of cultural sensitivity, privacy, and participant protection that remain and need to be addressed, regardless of the level of sophistication in an EDC system.

### Objectives

The objective of our paper is to present the design of an EDC system for the CARES trials and show how multiple EDC functions introduced above can be integrated into a single EDC system to support a large-scale behavioral intervention trial.

## Methods

### Adolescent Medicine Trials Network “Comprehensive Adolescent Research & Engagement Studies” Overview

A suite of interventions, “CARES,” is being conducted through the HIV ATN. Three separate studies share an overarching aim to address the increasing HIV epidemic among youth aged 12-24 years. Toward this goal, interventions were developed for YLH and HRY. Recruitment began in May 2017. Participants are being recruited through social service agencies, homeless shelters, HIV care clinics, and clinic referral in Los Angeles (LA) and New Orleans (NO). During recruitment, interested youth are screened, which seeds a case in the EDC. Eligible youth are consented and enrolled into one of three studies based on HIV test results and responses to screening questions. Enrolled youth are then administered a baseline assessment and are repeatedly assessed at 4-month intervals over a 2-year period. YLH are administered blood tests for HIV viral loads, and HRY are tested for HIV seropositivity. Both cohorts are tested for the presence of sexually transmitted infections (STI) and administered drug screenings and assessments to self-report on HIV-transmission risk behaviors.

Assignment to one of the following three studies occurs at enrollment.

- *Study 1 (ATN 147)* is an observational study and is enrolling YLH who are acutely infected with HIV or youth who are YLH but untreated with antiretroviral therapies (ART) or treatment naïve for comparison. ATN 147 participants are aggressively treated with highly potent ART, as soon as possible after infection to examine variation in viral reservoirs over time. Participants receive an Automated Messaging and Monitoring Intervention (AMMI). AMMI has two components. Participants receive daily SMS text messages on their mobile phones that are tailored to their specific health needs such as medication reminders and health messages that are appropriate for YLH. The second component administers a weekly SMS text message survey for self-management and to keep YLH engaged in the study; survey nonresponse prompts follow-up from study staff. In addition, participants receive peer support, through a Web-based platform, and coaching support. Coaching by trained paraprofessionals is provided to make treatment referrals and provide brief interventions.
- *Study 2 (ATN 148)* is a randomized controlled trial (RCT) and is enrolling YLH with an established and unsuppressed HIV infection. At enrollment, YLH are randomized to one of the two study arms—A Standard Care arm that provides the AMMI or a Stepped Care arm. In the Stepped Care arm, increasingly intense interventions are delivered if a participant’s viral load is detectable at follow-up visits. Steps of increasing care include the following: (1) the Standard Care AMMI; (2) standard care plus peer support; or (3) standard care plus peer support and coaching support.
- *Study 3 (ATN 149)* is an RCT and is enrolling HRY. Eligibility is based on being HIV negative and being at risk for acquiring HIV based on a number of risk factors. The HRY are randomized to one of four study arms in a factorial design—AMMI only as standard care (across arms), AMMI and online peer support, AMMI and coaching, or AMMI and online peer support with a coach. Similar to ATN 147 and 148, the weekly SMS text message survey provides an opportunity for self-management and engagement. In addition, the survey queries HRY on symptoms of HIV infection. Study staff follow-up with HRY indicating symptoms of acute HIV-related flu and referred for immediate HIV testing.

The EDC system was developed through the *CommCare* system, an open-source mobile data collection platform that is developed and hosted by *Dimagi, Inc*. In a typical research setting, a separate EDC system would have been developed for each study; we could have done so for ATN CARES using the *CommCare* system. A single recruitment strategy and assessment schedule across all three studies and in both cities facilitates the use of a single EDC system for most data collection and technology- based intervention activities. There is one exception to a single recruitment strategy that is being implemented across cities. We discuss the exception to highlight the flexibility of the EDC system to allow the study flow and data entry forms to differ by study and city if needed. Recruitment venues in NO include bars and other public venues that do not provide private spaces in which to administer the full screener. Instead, a (mini) prescreener is administered in public venues. Interested YLH or HRY who are eligible based on the prescreener are invited to be screened and enrolled, if eligible, in a more private setting (eg, a clinic). *CommCare* data collection includes screening, study assignment and randomization, enrollment, contact information, 4-month self-report assessments, laboratory test results (ie, HIV and STI tests, and drug screenings), and weekly SMS text message surveys. In addition, the CommCare system pushes automated SMS text messages to participants’ phones for intervention activities. After enrollment, participants receive an SMS text message on their mobile phone to welcome them to the study and inform them of their study arm assignment if they are enrolled in ATN 148 and 149. Furthermore, participants receive daily SMS text messages as part of the AMMI unless they opt out. All study procedures were approved by the institutional review boards at participating universities. Further details on each of the three studies may be found in the corresponding study protocol papers [23-25].

### Electronic Data Capture System Development for Adolescent Medicine Trials Network “Comprehensive Adolescent Research & Engagement Studies”

The development of the EDC system began with meetings between the ATN CARES research team and *Dimagi* technicians. Workflows were developed for all three studies described above and focused on major decision points regarding participant screening, enrollment, randomization, and retention. Determination of research staff roles in accessing the EDC system was carried out in parallel to the development of the workflow and is summarized in Table 1. Four roles were identified for the research staff who act as interviewers, coaches, study managers, or data managers.

**Figure 1:**
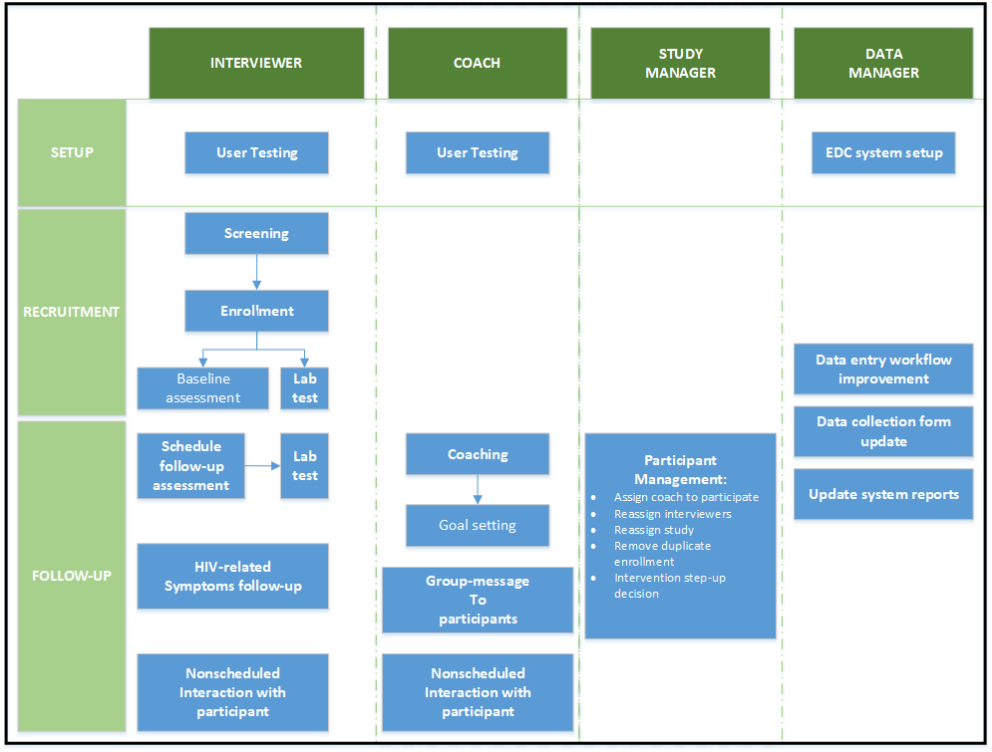
Schematic for roles of 4 types of research staff who access the electronic data capture (EDC) system and the study flow that pertains to each role during the study period.

Figure 1 provides an overview of the study workflow by role. In the figure, boxes without arrows connecting them to other boxes indicate processes that can occur at any time during setup, recruitment, or follow-up study phases. The EDC system was designed to allow data to be entered on the following study aspects: recruitment and retention, study intervention, study management, and outcome assessment. The EDC system offers flexibility in authorizing different levels of access to the study data depending on the role. Interviewers and coaches access the EDC system the most to enter and view study participant information across all three studies.

**Table 1:** Roles, purpose for accessing electronic data capture (EDC) system, and level of access granted for research staff who use the EDC system.

Role name	Access
Interviewer	Enter assessment, laboratory testing, and locator data via *CommCare* app. View participant data.
Coach	Enter semistructured coaching log via apps. Have access to reports and messaging utility, including sending out a group message to participants. View participant data.
Study manager	Complete research-related actions by submitting data such as rerandomization, reassigning a life coach for participants, or making stepped care decision. View reports and submitted data. Edit submitted data.
Data manager	Create and edit *CommCare* app. Create and edit reports and submitted data. Manage *CommCare* user access.

Both roles enable one to view the following key information for each participant: basic demographics, participant tracking information (eg, preferred methods of contact and contact information), session calendars that contain scheduled interviewer assessment dates or coaching session dates depending on the role, study participants’ progress in terms of completed assessments, and field notes that are entered by interviewers and coaches. Coaches have access to all notes, while interviewers have access only to interviewer notes.

Study managers utilize the EDC system to act upon a participant’s study status directly, such as investigator actively withdrawing a participant from the study, assigning a specific coach to a participant, or rerandomizing a participant if the HIV serum status changes. Study managers, coaches, and interviewers only have access to data collected in their city of residence (LA or NO). Restricted access not only adds a layer of protection for participant data but also streamlines research staff workflows. For example, coaches can more quickly locate, access, and browse participant notes than if they had to filter information from participants across both cities. The data manager has access to data across both cities. The data manager troubleshoots day-to-day data entry issues and can update any aspect of the EDC systems to reflect new study needs, including a change in assessment questionnaires and updating system reports or data entry workflow. In addition, the data manager can assist study managers in editing submitted data when data entry errors occur. Furthermore, the data manager creates reports for quality and progress monitoring of study teams.

#### Design of Electronic Data Collection Forms

Once the study flow was established, the research team worked with *Dimagi* technicians to design forms that supported data entry for each of the study tasks. Some forms were fairly standard based on prior HIV intervention trials that we had conducted and included assessment forms for in-person 4-month visits and weekly SMS surveys, laboratory forms to record laboratory testing results, and locator forms to enter participant contact and tracking information. A less standard form provided a place for coaches and interviewers to document nonscheduled visits with study participants, hereafter referred to as an *interaction log*.

Forms were initially developed outside of the EDC system for easy sharing and viewing by research team members, including members who would not be accessing the EDC system but gave input on its design. For example, the baseline and follow-up assessments were developed in Microsoft Word. Once finalized, forms were then built in the EDC system using one of the following three methods: (1) built directly within the EDC system by *Dimagi* technicians or the data manager; (2) built in Excel, converted to an XML file, and then uploaded into the EDC system as a readable XML file using a free Web-based service [26] (); or (3) built in Excel as a reference table (eg, SMS text message banks) and imported into the EDC system.

#### Electronic Data Capture System Testing

Prior to study initiation, study workflow was tested by conducting screening, enrollment, and baseline assessment procedures with mock study participants. This gave us a chance to ensure that the research staff understood how to use the EDC system, that questions and prompts on each form were properly specified, and that the EDC system was assigning participants to appropriate studies and randomizing when required. Mock interviews with study participants were supplemented by scenarios that were generated by the data manager to ensure that all possible scenarios were tested, even scenarios that were not anticipated to occur very often. For example, a scenario was generated for a YLH who was initially enrolled into ATN 149 but then tested HIV positive at a later date, requiring study reassignment and rerandomization if the youth was assigned to ATN 148. Testing was conducted in an iterative fashion where the data manager made corrections and enhancements to the EDC system specifications after each round of testing.

### Goal and Requirements of the Electronic Data Capture System

Several general system requirements were considered throughout the development phase for daily tasks across all three studies. The EDC system was designed to (1) integrate data entry tasks into a single EDC system across all three studies and both cities where the study is taking place (LA and NO); (2) be intuitive for user navigation so that minimum training for study staff would be required; (3) be easy to modify so that EDC system changes can be programmed in a timely manner by a trained data manager or other research staff member; (4) support and maintain both incoming and outgoing SMS text messages; and (5) offer high-level data security during data collection in the field and for data storage. More details on each system requirement are provided below.

### An Integrated System

Data across all three studies in LA and NO are entered and accessed through 2 points of access: a *CommCare* mobile app on an Android device, either a smartphone or computerized tablet, or a *CommCare* Web portal on a desktop computer. The primary goal in the development of the EDC system was to streamline study activities so that additional study data that needed to be captured outside of the EDC system were minimized, especially data on paper. An integrated and paperless system minimizes errors in transferring data between separate databases and allows real-time data synchronization and utilization.

Integration required the assignment of a unique participant identifier for each study participant in the EDC system across various data sources, such as survey, study management, and lab sample collection and storage. A participant is opened as a “case” within the EDC system and assigned a unique participant ID when they express interest in study participation, complete the screener, and are determined to be eligible based on the screening data. The same participant ID is then used for tracking lab specimen data and weekly survey responses. Integration was made possible by carefully planning all the decision points and data sources at the outset so that they could be captured in a single EDC system; EDC systems typically only collect study outcome data. The following paragraphs provide examples of study tasks and decision points that are often conducted outside of EDC systems but managed by the CARES EDC system.

#### Eligibility, Study Assignment, and Randomization

**Figure 2:**
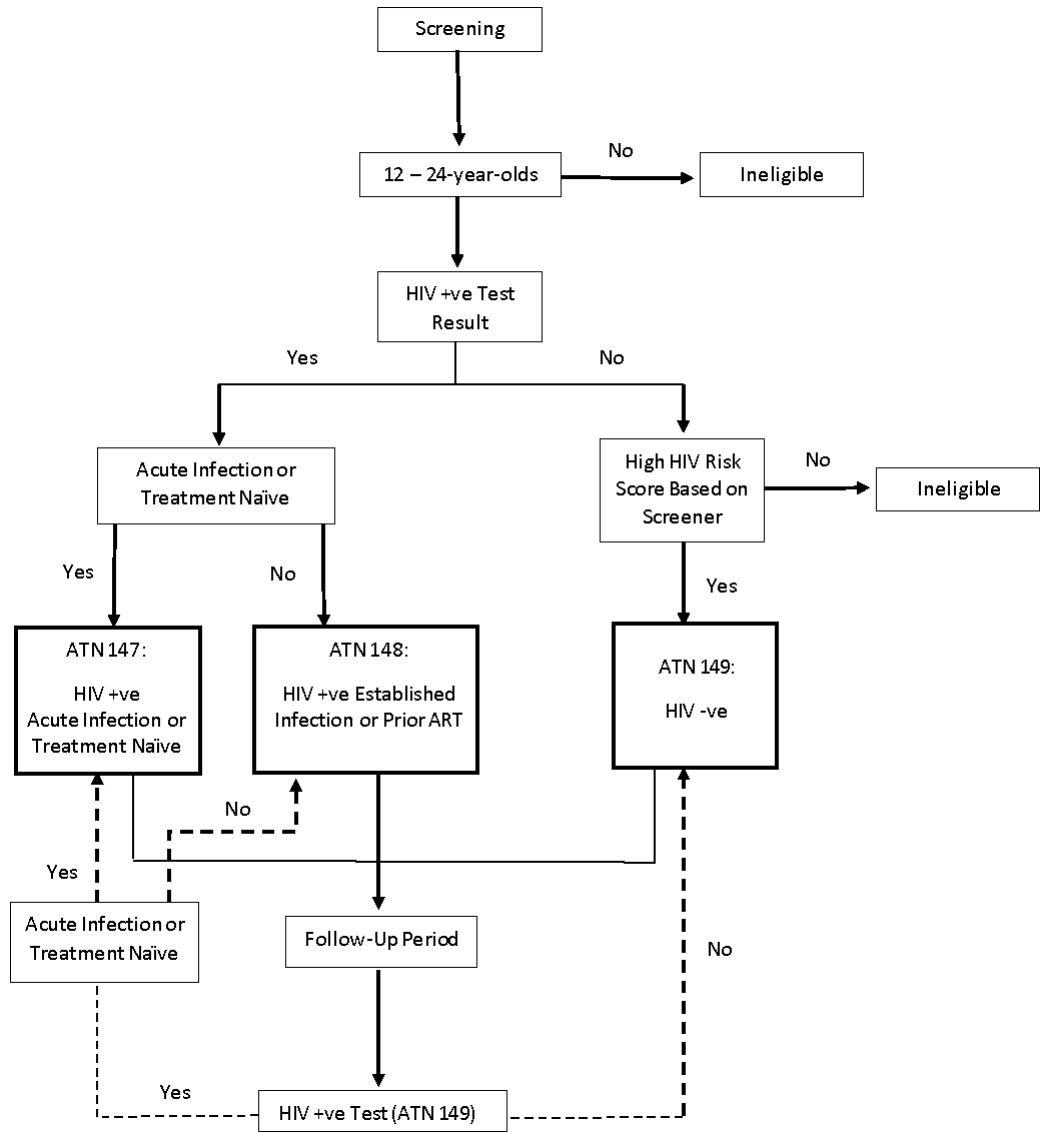
Study assignment schematic showing assignment to one of three studies (Adolescent Medicine Trials Network, ATN, 147-149) through the electronic data capture (EDC) system after screening; ATN 149 participants who test positive for HIV during the follow-up period are reassigned to ATN 147 or 148. ART: antiretroviral therapy.

Eligibility, study assignment, and randomization are all conducted within the EDC system. Figure 2 shows the decision processes. The first decision point for determining eligibility and study assignment is based on a rapid HIV testing information that is entered into the EDC laboratory form. A positive HIV diagnosis assigns participants to ATN 147 or 148. ATN 147 is recruiting acutely infected YLH and treatment-naïve YLH with established infections as a control sample. If a participant is treatment naïve, he or she will be preliminarily assigned to ATN 147. The ATN 147 laboratory team will then confirm through data entry the stage of infection based on various HIV RNA viral load blood tests. YLH with unsuppressed and established infections are recruited for randomization within ATN 148. A subsequent decision point occurs for interested youth who do not test HIV positive. They are eligible to participate in ATN 149 for HRY if the summation of responses to screening questions is above a certain threshold. For example, transgender youth and ethnic minority gay and bisexual youth automatically become eligible. Youth who identify as heterosexual but report multiple HIV risk factors such as homelessness, substance use, mental health problems, and past incarceration are classified as HRY and are eligible. Once participants are assigned to ATN 148 or 149, they are then randomized to a study arm.

#### Randomizations

ATN 148 and 149 are RCTs and require participants to be randomized after study enrollment is completed by entering rapid diagnostic test results. Study participants are automatically randomized to ATN 148 and 149 study arms within the EDC system. An exception occurs if study participants change studies and need to be randomized again. For example, it is possible for an HRY in ATN 149 to become HIV positive during the study (Figure 2). The participant would then be offered enrollment in ATN 147 or ATN 148 depending on the stage of infection and prior ART utilization. In this instance, interviewers update study assignment and data managers conduct the subsequent study arm (re)randomization.

Several randomization challenges were addressed so that randomization schemes could be programmed within the EDC system for ATN 148 and 149. RCT randomization schemes are typically stratified on one or more sociodemographic participant characteristics and site such as clinics. Randomization within the EDC system required careful consideration of how best to capture key variables and keep the scheme simple so that it could be programmed within the system. It was determined that the EDC system would feasibly accommodate 2 levels of stratification. Interviewer comprises the first stratification level and is a proxy for the site. An interviewer was used instead of site because the *CommCare* app is designed to function offline in the event of interrupted internet connectivity. This also means that randomization needs to occur through interviewers’ mobile phones without a connection to the EDC system. In general, interviewers are assigned to sites and serve as suitable site proxies. Self-reported sexual orientation identity (based on screening questions) was determined to be another important stratification variable and is being used as the second stratification variable for randomization.

#### Interaction Log

The interaction log records all nonscheduled interactions that any study staff have with participants, including content areas such as simple relationship building, participants updating contact information, rescheduling of participants’ appointments, confirmation of participants’ STI treatment, and so forth. For each interaction, we collect parameters that include date of the interaction and methods via which the interaction was completed (eg, phone, in-person, SMS text message, email, or social media). Specifically, we also require the study staff to report any failed interaction attempt to track staff effort required for each participant. This information is used for supervision and problem solving to maximize study retention.

#### Coach Monitoring Log

In addition to the interaction log, coaches record coaching activities through a separate log. This log records semistructured coaching activities, including coaching content areas and skills utilized, date of coaching, and the method of contact. The EDC systems record the length of each coaching session so that we can track coaching effort required for each participant assigned with a coach. Logged coaching information helps coaching supervisors to monitor and support coaching performance and better assign coaching workload. In addition, coaches are aided as they can refer to prior information through the mobile app to inform current interactions with participants. These data can also be used for analyses on engagement or utilization and dose-response effects.

#### Data Collected Outside of the Electronic Data Capture System

Despite efforts to capture data through a single EDC system, there are 3 instances where data are collected outside of the EDC system. First, participants were initially administered weekly surveys through SMS text messages. Response rates were low. Based on participant feedback, greater flexibility was provided to participants in filling out weekly surveys through SMS text message or an email prompt. Participants who receive email prompts are linked to a Web-based survey that is conducted through Research Electronic Data Capture. Second, participants who are randomized to receive peer support as an intervention component are asked to participate in a private social media discussion forum that is hosted through *Muut* [27], a JavaScript app. Conversation content data are collected through the *Muut* platform. Third, cost-effectiveness analyses will be conducted and require an estimation of staff time. Coaching logs and other intervention information are being entered into the EDC system, but they do not provide information regarding the amount of time spent performing study tasks. Staff activity information that is captured by the EDC system is being supplemented by *Time It*, a mobile app that allows individuals to record time spent carrying out different tasks.

### Intuitive Use

**Figure 3:**
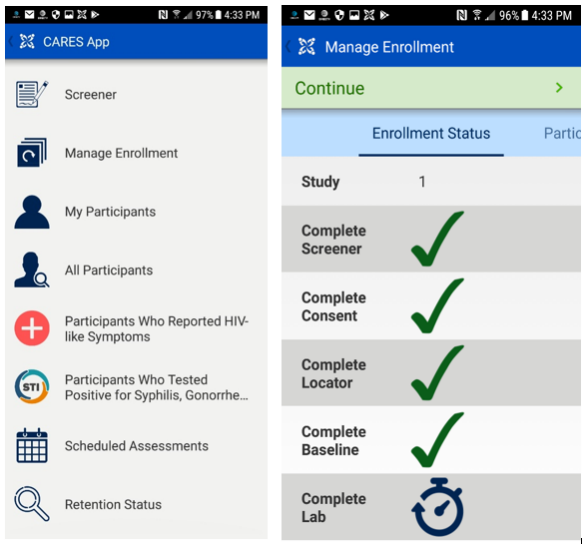
Screenshots of CommCare mobile app that links to the electronic data capture system.

The EDC system was developed to be intuitive to use to reduce user burden, minimize data entry error, and, in turn, reduce data cleaning. For data entry purposes, all EDC system users (shown in Table 1) access the system through a *CommCare* mobile app on an Android phone or tablet or through a Web portal on a computer. Figure 3 shows screenshots of the CommCare app that are seen by interviewers. The left-hand screenshot shows a menu with study tasks in a logical workflow that begins with “Screener” and ends with “Retention Status.” The right-hand screenshot shows the enrollment progress of a participant under the “Manage Enrollment” workflow indicated in the left-hand screenshot, with forms checked off once they are completed. The app was designed with an appealing dashboard that includes icons to guide users through their designated research tasks. The EDC system is set up to guide interviewers to complete the necessary steps for enrollment, including obtaining consent, collecting locator information, conducting a baseline assessment survey, as well as entering lab test data. Participants who complete the enrollment will then graduate to follow-up workflow, where (1) participants are randomized and may be opened up for life coaching interventions; (2) participants receive AMMI and surveys on their phone; and (3) follow-up assessments instead of baseline survey assessments will be available for those participants.

Intuitive use is also aided by built-in logic checks and data point validations. Users cannot enter information into subsequent fields within forms that clash with prior information that was entered into the system. For example, a warning will show up if an interviewer tries to enter an HIV viral load result for a seronegative participant. Information is also shared between forms; this means that information such as gender at birth and age that was collected during screening does not need to be asked again in the baseline assessment. Many participant characteristic variables such as HIV status and birth sex are used to set up appropriate skip patterns including for follow-up assessments. For example, questions regarding pregnancy at the baseline interview are only asked among female participants. HIV stigma questions are only asked among participants who had been positive for more than 4 months at the time of recruitment, that is, the baseline interview.

### Easy Modification

**Figure 4:**
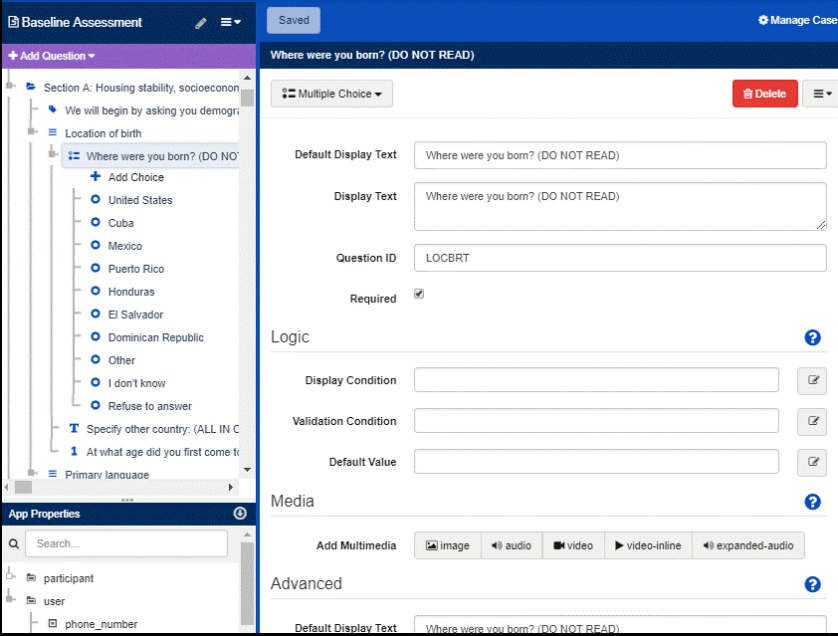
Screenshot of the electronic data capture system interface that is used by the data manager to create and edit assessment questions.

The EDC system is set up so that changes to any of the study forms can be programmed in a timely manner without any special programming abilities by a trained research staff member, mainly the data manager. Figure 4 illustrates the ability to easily modify forms. A screenshot is shown for one of the sociodemographic multiple-choice questions from the baseline assessment that queries place of birth. Fields such as “Display Text” are selected by mouse clicks and modified by keystrokes.

### Short Message Service Text Messaging

The EDC system was set up to manage all SMS text messaging with study participants. This occurs in 3 instances. Shortly after study enrollment, participants receive a welcome SMS text message that also contains information on intervention arm assignment, if participants are assigned to ATN 148 or 149. For example, if a participant is assigned to have a coach, both the participant and the coach receive an immediate SMS text message informing them of the initiation of their coaching relationship. Participants who are randomized to receive peer support are sent an SMS text message invitation to join a peer support group with a weblink to the registration page. All participants receive weekly SMS text message surveys and daily health promotion messages that span 5 domains as follows: general health, mental health, sexual health, substance abuse, and medication adherence. The specific messages that are sent to participants are based on one of four risk profiles that they are assigned to within the EDC system: gay, bisexual, and transgender youth (GBTY) living with HIV, non-GBTY who are living with HIV, high-risk GBTY, and high-risk non-GBTY.

### Data Security

The EDC system is set up to meet Web standards for compliance with the Health Insurance Portability and Accountability Act. The EDC system incorporates redundant safeguards to protect participant data. For example, mobile devices used for data entry are password-protected, saved form data are encrypted on the device and during transmission, and form submissions are transmitted and removed from devices as soon as internet connectivity is established.

## Results

### Experiences in Developing the Electronic Data Capture System

By using *CommCare* as the major EDC system, we were able to meet most of the requirements set during the study design. In particular, real-time data download allows generation of multiple reports that can inform recruitment planning, budgeting, life coaching quality, interviewer supervision, as well as data collection quality itself. In addition, the EDC specification that aims to minimize the need for participant tracking documents that reside outside of the EDC system improves their overall interaction experience with participants and increases field worker efficiency. For example, within the EDC system interviewer role, we can easily identify participants that require immediate follow-up efforts and, then, initiate contact with participants per their preference, such as their preferred way of contact and preferred pronoun that is used to address them in person or through an SMS text message. Finally, the EDC system is a nonprogrammer-friendly platform and allows nonprogramming data managers to update the EDC system for each study needs in a timely fashion. For example, as the field staff discover new types of interactions that they have with study participants, the data management team can easily expand and redefine the list of interactions available for selection within the interaction log to accommodate this unforeseen task in near real time.

### Electronic Data Capture System Implementation

At the time of writing, CARES studies have been in the field for approximately 10 months from May 2017 to early July 2018; recruitment is ongoing. We present sample sizes to summarize information that has been catalogued and managed through the EDC system and, in turn, to underscore the performance of the EDC system across studies 1-3. Of 1053 youth who have been screened (576 in LA and 477 in NO), 812 have been recruited and enrolled (408 in LA and 404 in NO). Study 1 enrolled 26 YLH, study 2 enrolled 70 YLH, and study 3 enrolled 716 HRY. Four-month assessments (ie, assessments at the first follow-up) have been conducted on 608 participants. In addition, the EDC system is being used to facilitate intervention delivery as discussed above. For example, a total of 16,994 daily SMS text messages have been sent to 627 participants’ phones, and 432 participants have filled out at least one weekly SMS text message survey as part of the AMMI. Among YLH in study 2 who have been randomized to the Stepped Care arm, 3 have stepped up from the AMMI to the next level of support.

### Electronic Data Capture System Challenges

We experienced several challenges after the implementation of the EDC system. Most of these challenges were resolved by modifying the system. We discovered multiple discrepancies between real-world field worker (ie, coach and interviewer) needs and workflow scenarios that the research team was able to envision and test before the EDC system was launched in the field. For example, the initial HIV clinical visit would be the ideal setting for the research staff to collect all baseline information for acute-infected YLH. However, the impact of learning of an HIV diagnosis during the first clinical visit is potentially overwhelming for YLH. It was decided that alternative study protocols should be set up so that research staff do not need to go through all the risk behavior questions listed in the screening form or any other required forms with newly diagnosed YLH during enrollment. As a result, we built additional features within the EDC system that allow interviewers to circumvent data entry into some of the enrollment forms that typically require data entry if an HIV-positive test result is also entered into the EDC system. A participant ID is still generated for lab specimen tracking. This alternative workflow allows interviewers to focus on the consent and blood draw with participants during their first clinical visit.

Despite the EDC system training for field workers prior to study implementation, the major challenges around the EDC system lie in data quality control among field workers. In particular, accurately logging ad-hoc participant interactions proved to be especially challenging for field workers. First, we found that field workers tend to prefer to enter information into the open-ended running notes as opposed to the prespecified types of interactions that are captured by form fields. Second, open-ended note documentation styles vary greatly among field workers, making a quick review of the notes across field workers harder. To address this, we first expanded the capabilities of the interaction log to capture additional data regarding field workers’ interactions with participants that we could not anticipate. Field workers were then required to memorize what information could be collected within the interaction log. Furthermore, the data management team put together additional data entry training sessions to standardize the use of open notes after consulting with the intervention team.

Another issue came up regarding daily SMS text messages and weekly surveys that the EDC system sends to participants’ phones. Participants can discontinue the receipt of SMS text messages and surveys during the study. Our original plan was that participants would have to contact the study staff to discontinue the receipt of daily messages and weekly surveys. We found that the SMS text message server is legally required by Federal Trade Commission regulations to allow a participant to opt out of all SMS text message services by replying to the daily message or weekly service with a “STOP” command. Obviously, those who opt out of the SMS text message intervention component will not receive the full intervention, essentially creating an additional ad-hoc intervention arm. Fortunately, the EDC system allows study managers to track the list of participants who opt out, and subsequently, field workers can reach out to study participants to attempt to re-engage them. In the least, the study team can track participants who self-select the ad-hoc intervention arm.

## Discussion

This paper describes an EDC system that is currently being used to support the implementation of a large-scale HIV intervention trial across multiple sites. Several key considerations underpinned the EDC system infrastructure development, and they should also be considered in the design of other EDC systems, regardless of study-specific requirements.

First, research teams need to decide whether EDC systems will be developed in-house or through a third-party vendor. The ATN CARES team chose the latter option to move away from the “pilotitis” paradigm, where a lot of money is spent on developing mHealth apps from the ground up that are not sustainable [28,29]. Instead, we are agnostic to specific technology with a focus on study procedures and intervention functions over specific technology platforms. For example, we focused on the development of the SMS text message content with the notion that multiple platforms can execute the messaging function of the intervention.

In the end, we decided to use the *CommCare* platform through Dimagi that used many “off-the-shelf” system components such that programming from the ground up would not be required. In addition, the *CommCare* system satisfies most of the requirements as described above and offers flexibility in determining costs on a fixed grant budget. We selected the *CommCare* PRO plan because it provides a basic level of technical support and training for the research team while also allowing us to reduce costs by having a trained research staff member to complete the majority of EDC system programming in-house, which saves cost in the long run. We selected *Dimagi* as our EDC system vendor based on prior lessons while working with numerous EDC system vendors. While cost and the ability to provide basic system requirements are important considerations, we strongly advise selecting vendors based on prior experience collaborating on research projects. Basic system requirements that are intrinsic to research, such as randomization and the need to protect patient data, are not as likely to be obvious to vendors without prior research collaborations and are likely to result in more system development iterations than what would be required by working with a research-oriented vendor. Additional time and effort can easily offset what seemed to be initial cost savings.

Second, the needs of the target population and the protection of electronic data collected on the target population were the top priorities in the development of the EDC system. A number of electronic safeguards were put in place to ensure a high degree of security as described in the Methods section. In addition to electronic safeguards, there is no substitute for the formative work that precedes the implementation of any behavioral intervention trial. In an iterative fashion, the CARES research team discussed all study procedures and presented study protocols to community advisory boards that comprised the target population, prior to the development of the EDC system. The SMS text message content, for example, was highly vetted and pilot-tested by the CARES research staff prior to the implementation of the study. A priority was placed on developing the SMS text message content that is culturally sensitive and would not contain any specific references to HIV; medication reminders were generic to encourage general medication adherence.

A limitation of the EDC system is that it does not support all data entry tasks and technology functions that the intervention requires. For example, the EDC system does not support online peer support groups; a separate system was set up through *Muut* for that purpose as discussed earlier. We posit that the inability to rely on any single EDC system to meet all study requirements is likely to be the case for a large-scale intervention trial. In addition, we note that the use of different technology solutions that support EDC, Web-based discussions, email, and other functions mirrors how we use different social media tools to meet different needs in our daily routines. These different options add to the richness of possibilities for the development of future behavioral intervention trials.

The EDC system that was developed for the ATN CARES trial presents as an example of how a commercially available and integrated data capturing system can meet all system requirements and support almost all needs of a large-scale research trial. While most EDC challenges can be resolved through programming updates, there are important considerations prior to the database implementation, such as identifying an appropriate EDC vendor with research experience and consulting with the target study population in developing participant-friendly study content.

## Abbreviations

AMMI: Automated Messaging and Monitoring Intervention
ART: antiretroviral therapies
ATN: Adolescent Medicine Trials Network
CARES: Comprehensive Adolescent Research & Engagement Studies
EDC: electronic data capture
EMA: ecological momentary assessment
GBTY: gay, bisexual, and transgender youth
HRY: high-risk youth
LA: Los Angeles
NO: New Orleans
RCT: randomized controlled trial
SMS: short message service
STI: sexually transmitted infections
YLH: youth living with HIV
